# STAGING LAPAROSCOPY IS STILL A VALUABLE TOOL FOR OPTIMAL GASTRIC CANCER MANAGEMENT

**DOI:** 10.1590/0102-672020220002e1700

**Published:** 2023-01-09

**Authors:** Erica SAKAMOTO, Marcus Fernando Kodama Pertille RAMOS, Marina Alessandra PEREIRA, André Roncon DIAS, Ulysses RIBEIRO, Bruno ZILBERSTEIN, Sergio Carlos NAHAS

**Affiliations:** 1 Universidade de São Paulo, University Hospital, Department of Gastroenterology of Faculty of Medicine – São Paulo (SP), Brazil.

**Keywords:** Stomach Neoplasms, Laparoscopy, Neoplasm Staging, Peritoneal Neoplasms, Neoplasias Gástricas, Laparoscopia, Estadiamento de Neoplasias, Neoplasias Peritoneais

## Abstract

**BACKGROUND:**

Complete surgical resection is the main determining factor in the survival of advanced gastric cancer patients, but is not indicated in metastatic disease. The peritoneum is a common site of metastasis and preoperative imaging techniques still fail to detect it.

**AIM:**

The aim of this study was to evaluate the role of staging laparoscopy in the staging of advanced gastric cancer patients in a Western tertiary cancer center.

**METHODS:**

A total of 130 patients with gastric adenocarcinoma who underwent staging laparoscopy from 2009 to 2020 were evaluated from a prospective database. Clinicopathological characteristics were analyzed to identify factors associated with the presence of peritoneal metastasis and were also evaluated the accuracy and strength of agreement between computed tomography and staging laparoscopy in detecting peritoneal metastasis and the change in treatment strategy after the procedure.

**RESULTS:**

The peritoneal metastasis was identified in 66 (50.76%) patients. The sensitivity, specificity, and accuracy of computed tomography in detecting peritoneal metastasis were 51.5, 87.5, and 69.2%, respectively. According to the Kappa coefficient, the concordance between staging laparoscopy and computed tomography was 38.8%. In multivariate analysis, ascites (p=0.001) and suspected peritoneal metastasis on computed tomography (p=0.007) were statistically correlated with peritoneal metastasis. In 40 (30.8%) patients, staging and treatment plans changed after staging laparoscopy (32 patients avoided unnecessary laparotomy, and 8 patients, who were previously considered stage IVb by computed tomography, were referred to surgical treatment).

**CONCLUSION:**

The staging laparoscopy demonstrated an important role in the diagnosis of peritoneal metastasis, even with current advances in imaging techniques.

## RESUMO

**RACIONAL:**

A ressecção cirúrgica é o principal fator determinante na sobrevida de pacientes com câncer gástrico, mas não é indicada na presença de doença metastática. O peritônio é local comum de metástase, porém os métodos de imagem ainda falham na sua detecção.

**OBJETIVO:**

Avaliar o papel da Laparoscopia Diagnóstica no estadiamento de pacientes com câncer gástrico avançado em um centro oncológico ocidental terciário.

**MÉTODOS:**

Foram avaliados 130 pacientes com adenocarcinoma gástrico submetidos a Laparoscopia Diagnóstica de 2009 a 2020, a partir de um banco de dados prospectivo. As características clínico-patológicas foram analisadas para identificar fatores associados à presença de metástase peritoneal. Foram também avaliadas a acurácia e concordância entre a tomografia computadorizada e a Laparoscopia Diagnóstica na detecção de metástase peritoneal e na mudança de conduta após a Laparoscopia Diagnóstica.

**RESULTADOS:**

As metástases peritoneais foram identificadas em 66 pacientes (50,76%). A sensibilidade, especificidade e acurácia da tomografia computadorizada na sua detecção foram de 51,5%, 87,5% e 69,2%, respectivamente. De acordo com o coeficiente Kappa, a concordância entre a Laparoscopia Diagnóstica e a tomografia computadorizada foi de 38,8%. Na análise multivariada, ascite (p=0,001) e suspeita de metástase peritoneal na tomografia computadorizada (p=0,007) foram estatisticamente correlacionadas com metástase peritoneal. Em 40 pacientes (30,8%), o estadiamento e as estratégias de tratamento mudaram após a Laparoscopia Diagóstica (32 pacientes evitaram laparotomia e 8 pacientes, anteriormente considerados estágio IVb, foram tratados cirurgicamente).

**CONCLUSÕES:**

A Laparoscopia Diagnóstica demonstrou um papel importante no diagnóstico de metástases peritoneais, mesmo com métodos de imagem avançados.

## INTRODUCTION

Gastric cancer (GC) is the third leading cause of death in the world^5^. The high incidence rates of GC in East Asian countries led to the adoption of screening strategies such as upper digestive endoscopy, which results in early diagnosis. In contrast, in Western countries, most cases are diagnosed in advanced stages. In Brazil, patients with advanced disease represent around 85% of all GC cases^26,27^.

Surgery remains the main curative treatment option for advanced gastric cancer (AGC), and gastrectomy with D2 lymphadenectomy is the standard surgical treatment. However, approximately 15% of patients have peritoneal metastasis (PM) at the diagnosis^16^.

Clinical staging includes upper digestive endoscopy and computed tomography (CT) scan of the chest, abdomen, and pelvis. However, the accuracy of CT scan to diagnose PM is low^6^. Previous studies have demonstrated that CT sensitivity for detecting PM ranges from 14 to 59%^19^. In many cases, PM is only detected during laparotomy, making the indication of gastrectomy questionable. In these situations, chemotherapy is the standard treatment if the patients are asymptomatic. REGATTA trial demonstrated that surgical resection followed by chemotherapy does not provide any survival benefit for incurable AGC compared with palliative chemotherapy alone^8^. Thus, surgical resection in those cases only adds risks of postsurgical complications and delays the onset of chemotherapy^9^.

Staging laparoscopy (SL) in GC was introduced in the early 1980s^11^. It allows detailed inspection of the entire peritoneal surface, collection of oncotic cytology, and identification of suspicious lesions and biopsies. It can be used both to confirm suspected peritoneal lesions on imaging studies and to rule out carcinomatosis prior to neoadjuvant treatment^7,9^. Several studies have demonstrated its superiority regarding conventional imaging tests to detect PM. In addition, SL is a low-invasive treatment with a low risk of postoperative complications^13,15^.

However, some discrepancies regarding which patients it should be performed and its importance in defining the therapeutic approach are still the subjects of discussion.

The objective of this study was to evaluate the role of SL in GC patients in a single Western tertiary cancer center. In addition, the accuracy of SL in relation to CT scan findings was also evaluated.

## METHODS

All patients with GC who underwent SL from 2009 to 2020 were evaluated from a prospective database. Only patients with gastric adenocarcinoma were included. Indications for SL were AGC (T3, T4), suspected peritoneal lesions, and staging prior to neoadjuvant chemotherapy. Laparoscopies performed for other purposes such as inflammatory acute abdomen, evaluation for recurrence, and resectability after chemotherapy were excluded.

Patients were staged preoperatively through abdominal and pelvis CT scans, upper digestive endoscopy, and laboratory tests. Clinical characteristics evaluated included age, sex, body mass index (BMI), preoperative laboratory tests (serum hemoglobin, serum albumin, neutrophil-to-lymphocyte ratio [NLR]^24^), and the presence of comorbidities using the Charlson Comorbidity Index (CCI) and the American Society of Anesthesiologists (ASA) classification.

The location of the tumor (classified as cardia, fundus, body, antrum, or whole stomach), macroscopic configuration (using the JGCA classification, which is based on Borrmann classification), and tumor size were assessed by endoscopic examination.

CT radiological reports were used for tumor staging. CT criteria for staging were based on previous studies^18^ and included the following parameters according to the TNM 8th edition^2^.

- For depth invasion (T):
  T1: Focal thickening/enhancement of the inner layer
  T2: Complete thickening of the gastric wall, with loss or disruption of a low-attenuation stripe at the base of the lesion, but a clear and well-defined outer gastric surface
  T3: Complete thickening of the gastric wall, with visually impossible discrimination between the gastric lesion and the outer layer, perigastric fat tissue preserved.
  T4a: Complete thickening of the gastric wall, with irregular or nodular outer layer and perigastric fat infiltration.
  T4b: Infiltration of adjacent organs.
- Lymph nodes of size greater than 10 mm in the short-axis diameter were considered malignant and recorded as absent or present.
- Presence of ascites, signs suggestive of PM (e.g., peritoneal thickening/enhancement, peritoneal nodules, or presence of omental cake), liver metastasis, or distant lymph node metastasis (retropancreatic, pancreaticoduodenal, peripancreatic, superior mesenteric, middle colic, para-aortic, or retroperitoneal node) were evaluated.

The postoperative follow-up was performed quarterly in the first year and every 6 months in the following years. Follow-up tests for relapse detection were based on the presence of symptoms. The absence of appointments for more than 12 months was considered a loss of follow-up. All cases were operated in a high-volume center by specialized surgeons.

The study was approved by the Hospital’s Ethics Committee and registered online (https://plataformabrasil.saude.gov.br, CAAE 25512819.1.0000.0065).

### Staging Laparoscopy

Staging laparoscopy was performed according to the standard technique used at our institution with the patient under general anesthesia. The first 10 mm trocar was placed in the supraumbilical region and a 12 mmHg pneumoperitoneum was established. Two 5 mm trocars were placed in the right and left lateral abdomen. The peritoneal cavity was inspected with careful investigation of the stomach surface, liver, diaphragm, peritoneum, omentum, mesentery, small bowel, and pelvic organs. The omental bursa was also inspected in cases of high suspicion of involvement (tumors located on the posterior wall of the stomach). Biopsies were performed for suspicious lesions and sent for pathological examination.

### Statistical Analysis

The chi-square tests were used for categorical variables and t-tests for continuous variables. The association of clinical characteristics with the diagnosis of peritoneal carcinomatosis by SL was analyzed by binary logistic regression analysis, and odds ratios (ORs) with a 95% confidence interval (95%CI) were calculated. Significant variables were included in the multivariate model.

The agreement between SL and CT was evaluated using the Kappa statistic. The Kappa coefficient was interpreted according to the Landis and Koch criteria and classified as poor (<1%), slight (1–20%), fair (21–40%), moderate (41–60%), substantial (61–80%), and almost perfect (81–100%).

Overall survival (OS) was estimated using the Kaplan-Meier method, and differences in survival were examined using the log-rank test. Survival time (months) was calculated from the date of surgery until the date of death. The patients who were alive were censored at the date of the last contact. All tests were two-tailed, and p<0.05 was considered statistically significant. The analysis was performed using the SPSS software, version 18.0 (SPSS Inc., Chicago, IL, USA).

## RESULTS

### Clinicopathological Characteristics

**Figure 1.**
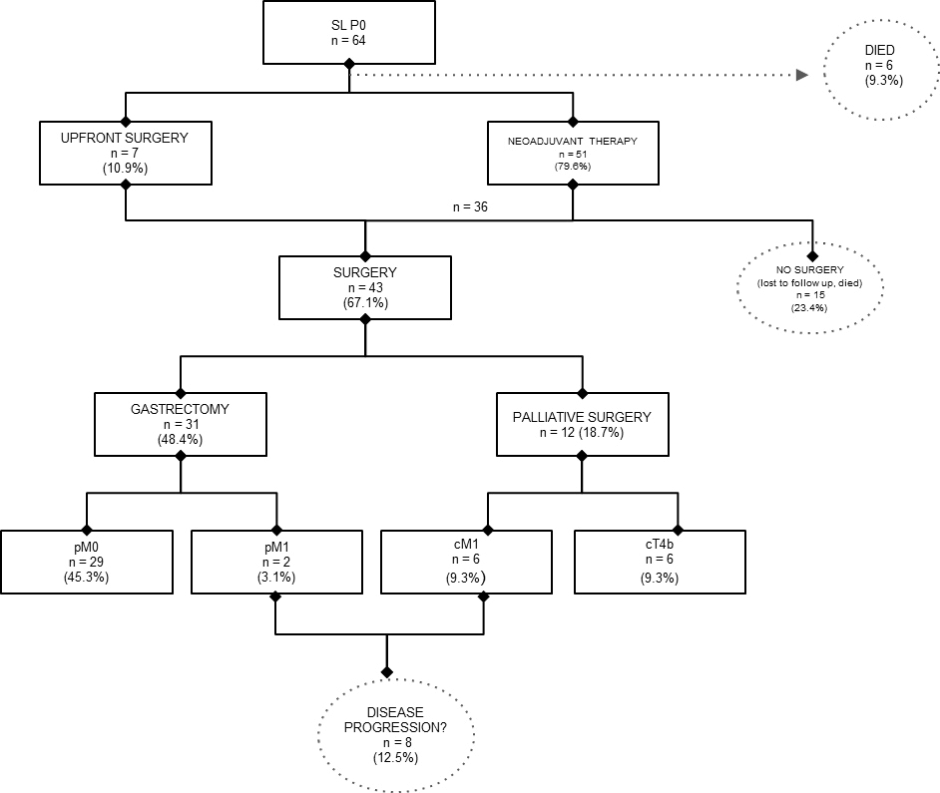
Treatment after staging laparoscopy.

SL: staging laparoscopy; P0: without peritoneal disease.

In the study period, a total of 130 GC patients who underwent SL were included in this study (Figure 1). The mean age was 59.6 years (range 24–86 years), with a mean BMI of 22.5 kg/m^2^ and male predominance (63.1%). The majority of patients had cT4 (80%) lesions, positive LN metastasis (85.5%), and no suspected distant metastasis (64.6%). The characteristics of the patients are presented in Table 1.

**Table 1.** Characteristics of gastric cancer patients who underwent staging laparoscopy.

Variables	n=130	%
Sex		
Female	48	36.9
Male	82	63.1
Age (years)		
Mean (SD)	59.6 (13.8)	
Range	24 - 86.6	
BMI (kg/m^2^)		
Mean (SD)	22.5 (4.8)	
Albumin (g/dL)		
Mean (SD)	3.7 (0.6)	
Hemoglobin (g/dL)		
Mean (SD)	11.2 (2.1)	
Neutrophil-to-lymphocyte ratio (NLR)		
Mean (SD)	4.03 (4.9)	
Charlson-Deyo Comorbidity Index (CCI)		
0	97	74.6
≥1	33	25.4
ASA (American Society of Anesthesiologists)		
I/II	86	66.2
III/IV	44	33.8
cT		
T1	1	0.8
T2	6	4.6
T3	19	14.6
T4a	56	43.1
T4b	48	36.9
Status cT		
≤T3	26	20.0
T4a	56	43.1
T4b	48	36.9
cN		
N0	19	14.6
N1	21	16.2
N2	50	38.5
N3	40	30.8
cM		
cM0	84	64.6
cM1	46	35.4
Staging		
I	1	0.8
IIA	7	5.4
IIB	6	4.6
III	42	32.3
IVA	27	20.8
IVB	47	36.2
Presence of ascites on imaging		
Absent	71	54.6
Present	59	45.4
Peritoneal lesions on imaging		
No	88	67.7
Yes	42	32.3
*Linitis plastica*		
Absent	110	84.6
Present	20	15.4
Tumor site		
Antrum/body	80	61.5
Fundus/cardia	33	25.4
Whole organ	17	13.1
Remnant gastric cancer		
No	122	93.8
Yes	8	6.2
Gastric wall		
Circumferential	73	56.2
Greater curvature	7	5.4
Lesser curvature	39	30.0
Anterior wall	3	2.3
Posterior wall	8	6.2
Tumor size (cm)		
Mean (SD)	7.3 (3.3)	
Stenosis		
Absent	94	72.3
Present	36	27.7
Macroscopic type		
I	2	1.5
II	8	6.2
III	88	67.7
IV	27	20.8
V	5	3.8
Lauren type		
Intestinal adenocarcinoma	42	32.3
Diffuse/mixed adenocarcinoma	79	60.8
Adenocarcinoma (not specified)	9	6.9

BMI: body mass index.

**Figure 2.**
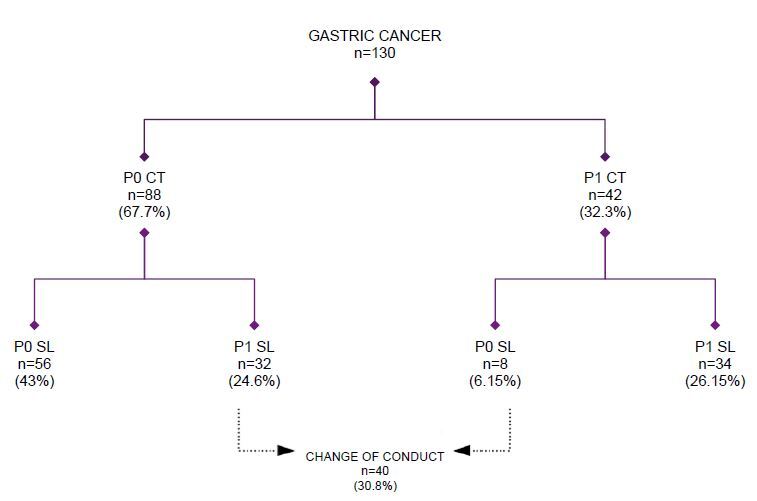
Change in treatment strategy after staging laparoscopy.

CT: computed tomography; SL: staging laparoscopy; P0: without peritoneal disease; P1: peritoneal disease.

Based on the SL result, patients were divided into two groups: P1 group, represented by 66 (50.76%) patients with evidence of peritoneal disease, and P0 group, represented by 64 (49.23%) patients with no evidence of peritoneal disease (Figure 2).

Clinical and pathological characteristics according to the diagnosis of carcinomatosis (P1 and P0 groups) are presented in Table 2. Patients in the P1 group were more likely to have circumferential tumors (p=0.005), to have linitis plastica (p=0.001), and to be classified as Lauren’s diffuse type (p=0.001). Tumor stenosis was observed in 25 and 30.3% of patients in P0 and P1 groups, respectively (p=0.499). A total of 3 (4.7%) patients in the P0 group and 5 (7.6%) patients in the P1 group had remnant gastric cancer (p=0.493). There were no differences in age, sex, BMI, the presence of comorbidities, and laboratory tests between groups.

**Table 2.** Clinical characteristics of gastric cancer patients according to the diagnosis of carcinomatosis by staging laparoscopy.

Variables	P0 SL	P1 SL	p-value
	n=64 (%)	n=66 (%)	
Sex			
Female	22 (34.4)	26 (39.4)	0.553
Male	42 (65.6)	40 (60.6)	
Age (years)			
Mean (SD)	61.6 (11.9)	57.5 (15.3)	0.092
BMI (kg/m^2^)			
Mean (SD)	22.6 (4.8)	22.3 (4.8)	0.719
Albumin (g/dL)			
Mean (SD)	3.8 (0.5)	3.6 (0.6)	0.100
Hemoglobin (g/dL)			
Mean (SD)	10.9 (2.1)	11.4 (2.1)	0.291
Charlson-Deyo Comorbidity Index (CCI)			
0	47 (73.4)	50 (75.8)	0.761
≥1	17 (26.6)	16 (24.2)	
ASA (American Society of Anesthesiologists)			
I/II	44 (68.8)	42 (63.6)	0.538
III/IV	20 (31.2)	24 (36.4)	
Circumferential lesion			
Yes	28 (43.8)	45 (68.2)	**0.005**
No	36 (56.2)	21 (31.8)	
Tumor size (cm)			
Mean (SD)	7.0 (3.2)	7.8 (3.4)	0.210
*Linitis plastica*			
Absent	61 (95.3)	49 (74.2)	**0.001**
Present	3 (4.7)	17 (25.8)	
Tumor site			
Antrum/body	44 (68.8)	36 (54.6)	**0.004**
Fundus/cardia	18 (28.1)	15 (22.7)	
Whole organ	2 (3.1)	15 (22.7)	
Lauren type			
Intestinal	31 (48.4)	11 (16.7)	**0.001**
Diffuse/mixed	29 (45.3)	50 (75.8)	
Adenocarcinoma (not specified)	4 (6.2)	5 (7.6)	
Macroscopic type			
I	2 (3.1)	0 (0)	**0.001**
II	6 (9.4)	2 (3)	
III	49 (76.6)	39 (59.1)	
IV	6 (9.4)	21 (31.8)	
V	1 (1.6)	4 (6.1)	

Bold indicates significant value. BMI: body mass index; SD: standard deviation.

### CT Staging and Imaging Analysis

The majority of patients in both groups (P0 and P1) were classified as cT4 and had positive lymph node metastasis (cN+). Table 2 presents CT scan findings (TNM staging, presence of ascites, signs suggestive of PM, liver metastasis, or lymph node involvement) stratified by peritoneal disease status. The presence of PM, ascites, and stage cIVb on CT scan was associated with P1 (p<0.05). Neither lymph node nor liver metastasis was significantly associated with P1.

### Accuracy and Strength of Agreement Between CT and SL in Detecting PM

Of the 130 patients who underwent SL, 42 were classified by CT as positive for PM (P1 CT). Among them, 34 confirmed the presence of PM on subsequent SL (positive predictive value [PPV], 81%) (Figure 1).

In the remaining 88 patients classified by CT as negative for PM (P0 CT), SL confirmed the absence of PM in 56 patients (negative predictive value [NPV], 63.6%).

The sensitivity, specificity, and diagnostic accuracy calculations for CT detection of PM are presented in Table 3. Using SL results as a reference, the sensitivity and specificity of CT were 51.5 and 87.5%, respectively. According to the Kappa coefficient, the concordance between SL and CT in the diagnosis of carcinomatosis was 38.8%.

**Table 3.** Computed tomography diagnosis and outcomes of gastric cancer patients according to the diagnosis of carcinomatosis by staging laparoscopy.

Variables	P0 SL	P1 SL	p-value
	n=64 (%)	n=66 (%)	
Presence of ascites on CT			
Absent	51 (79.7)	20 (30.3)	**<0.001**
Present	13 (20.3)	46 (69.7)	
Peritoneal lesions on CT			
No	56 (87.5)	32 (48.5)	**<0.001**
Yes	8 (12.5)	34 (51.5)	
Liver metastasis on CT			
No	64 (100)	64 (97)	0.496
Yes	0 (0)	2 (3)	
cT			
≤cT3	17 (26.6)	9 (13.6)	0.147
cT4a	27 (42.2)	29 (43.9)	
cT4b	20 (31.2)	28 (42.4)	
cN			
cN0	9 (14.1)	10 (15.2)	0.861
cN+	55 (85.9)	56 (84.8)	
cM			
M0	56 (87.5)	28 (42.4)	**<0.001**
M1	8 (12.5)	38 (57.6)	
cTNM			
≤II	10 (15.6)	4 (6.1)	**<0.001**
III/IVA	46 (71.9)	23 (34.8)	
IVB	8 (12.5)	39 (59.1)	

Bold indicates significant value. CT: computed tomography; SL: staging laparoscopy; P0: without peritoneal disease; P1: peritoneal disease.

The analysis of risk factors for the diagnosis of carcinomatosis by SL demonstrated that ascites (p=0.001) and suspected PM on the CT scan (p=0.007) were statistically correlated with the P1 group in multivariate analysis (Table 4).

**Table 4.** Comparison between staging laparoscopy and computed tomography staging for carcinomatosis (P1).

Group	P1 SL	P0 SL	***Total***	Predictive values
P1 CT	34	8	*42*	34/42—PPV 81.0%
P0 CT	32	56	*88*	56/88—NPV 63.6%
Total	*66*	*64*	*130*	
	Sensitivity—34/66 (51.5%)	Specificity—56/64 (87.5%)		Accuracy 90/130 (69.2%)

SL: staging laparoscopy; CT: computed tomography; P1: peritoneal metastasis; PPV: positive predictive value; NPV: negative predictive value. P0: peritoneal disease

### SL and Treatment Decision-Making

**Figure 3.**
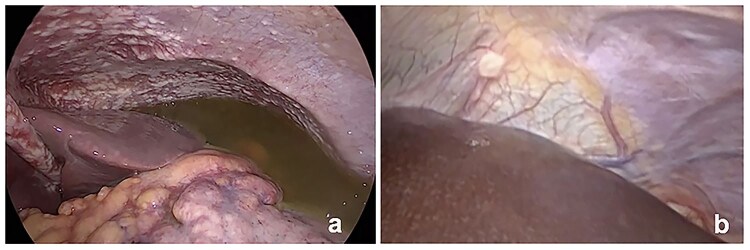
Staging laparoscopy with macroscopic carcinomatosis. (a) Gross peritoneal spread; (b) Single node.

Among the 130 patients who underwent SL, 88 (67.7%) patients had no signs of PM on CT scan (P0 CT). Of these patients, 32 (24.6%) were found to have positive occult peritoneal metastatic disease on SL and were able to avoid unnecessary laparotomy (Figure 3). In contrast, of the 42 (32.3%) patients who had suspected PM on CT scan (P1 CT), SL excluded PM in 8 (6.15%) patients. Thus, in 40 (30.8%) patients, staging and treatment plans changed after SL (Figure 1).

### Treatment After SL

Of the 64 patients classified as P0 after SL, 51 (79.6%) were referred to neoadjuvant chemotherapy and 7 (10.9%) patients to upfront surgery. In total, 43 (67.1%) patients underwent surgical treatment. A total of 31 (48.4%) patients underwent gastrectomy with curative intent, and in two patients, the anatomopathological results of the surgical specimen revealed PM. Another 12 (18.7%) patients had criteria for nonresectability in the second surgery and underwent palliative surgery (6 patients due to PM and 6 patients due to invasion of adjacent structures). A total of 8 (12.5%) patients had PM at the second surgery.

### Overall Survival

When comparing GC patients according to the M status by SL (M1 SL vs. M0 SL), the survival of patients with peritoneal carcinomatosis was significantly worse than those without carcinomatosis (7.5 vs. 16.5 months, p<0.001) (Table 5).

**Table 5.** Univariate and multivariate analysis of factors associated with the diagnosis of carcinomatosis by staging laparoscopy (P1 group).

Variables*	Univariate		p-value	Multivariate		p-value
	OR	95%CI		OR	95%CI	
Age ≥ 65 (vs. <65 years)	0.89	0.44–1.79	0.746	─	─	─
Male (vs. female)	0.81	0.39–1.65	0.554	─	─	─
Proximal/whole (vs. antrum/body)	1.81	0.89–3.75	0.098	─	─	─
Circumferential (vs. non-circumferential)	2.75	1.35–5.64	**0.006**	1.06	0.42–2.67	0.898
*Linitis plastica* (vs. absent)	7.05	1.95–25.47	**0.003**	3.21	0.70–14.65	0.132
cT4 (vs. others)	2.29	0.94–5.61	0,070	─	─	─
cN+ (vs. cN0)	0.92	0.35–2.43	0,861	─	─	─
P1 CT (vs. P0)	7.44	3.07–18.01	**<0.001**	3.96	1.45–10.81	**0.007**
Ascites (vs. absent)	9.02	4.04–20.16	**<0.001**	4.94	1.98–12.35	**0.001**
Diffuse/mixed (vs. other)	3.77	1.79–7.97	**0.001**	2.45	0.99–6.05	0.052

Bold indicates significant value. OR: odds ratio; CI: confidence interval; P: Peritoneum; CT: computed tomography. P1: peritoneal disease.

**Figure 4.**
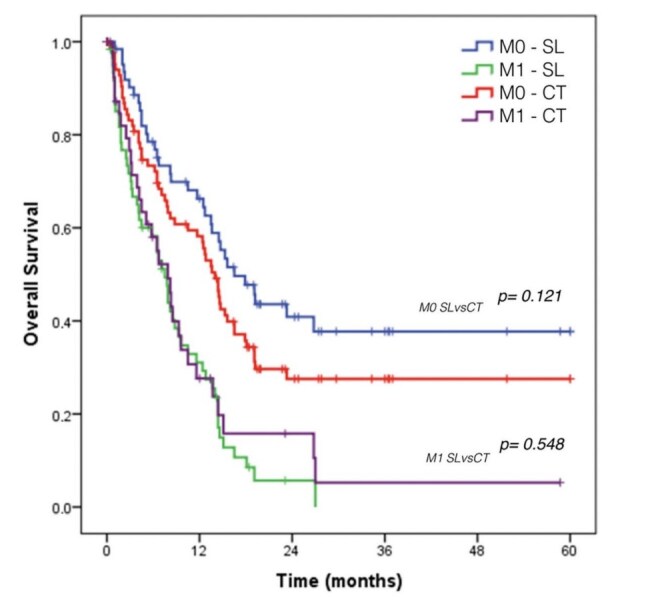
Overall survival of gastric cancer patients according to the M status by computed tomography and staging laparoscopy.

CT: computed tomography; SL: staging laparoscopy.

When comparing M0 GC according to the method of staging, the mean OS for M0 patients who underwent SL was superior to patients who did not (16.5 vs. 14 months) but without significance (p=0.121) (Figure 4).

## DISCUSSION

Although surgery represents the cornerstone of AGC treatment, it is mainly indicated in the absence of metastatic disease. Despite the proven value of SL in the diagnosis of PM, there is no consensus yet on its routine use in clinical practice, and it remains underutilized in the management of GC, with reported frequencies of less than 25%^4,10^. Thus, the present study was developed to further address this issue and to highlight the importance of SL in the diagnosis of PM and treatment strategy. In our study, of the 130 patients who underwent SL, PM was found in 66 (50.76%). In addition, our results regarding the accuracy of CT scan were consistent with previous studies and confirmed its limitations in detecting PM, with 51.5% of sensitivity, 87.5% of specificity, and 69.2% of accuracy**.**

Even with technological advances in imaging diagnostics, SL still provides a superior ability to inspect the peritoneal surface. Currently, several guidelines include its use in staging^1,3^. The main objective is to detect occult PM and other factors that can change the therapeutic strategy, such as the invasion of adjacent structures and liver metastasis^9^. However, the appropriate selection of patients who are candidates for SL is still controversial and differs among various institutions. Some recommend it for all resectable GC (stage IB-III), especially for those who are being considered for neoadjuvant treatment^30^.

Based on previous literature, it is difficult to define the most appropriate indications for SL, notably due to the differences in study populations and inclusion criteria. In the Western series, where most cases are diagnosed in advanced stages, the tendency was to perform it in all patients with AGC. While in the Eastern series, where most patients present with early-stage disease, the recommendation was to perform it selectively in individuals at high risk for PM^15,25,31^. A systematic review carried out by Fukugawa et al. found that, in Japanese institutions, SL evidenced positive findings in 42.7–53.4% of the cases, higher than in other countries (7.8–40%). This discrepancy was partly because they had a greater selection of patients at risk for PM^9^. In our study, PM was found in 66 (50.8%) patients who underwent SL, which is also a higher number compared to previous studies, mainly due to the same selection bias of high-risk cases.

Some factors are known to increase the poor prognosis of GC and to be related to the presence of PM, such as large Bormann type 3 and Bormann type 4^12,13,14^. In a prospective cohort study, with prespecified indications for SL, Irino et al. found a greater impact, achieving an accuracy of 91.5%. Their indications were large = 8 cm Borrmann type 3, Borrmann type 4, bulky lymph nodes or para-aortic lymph node involvement, and suspicion of PM on CT scan^15^. In our study, the presence of ascites (p=0.001) and suspected PM on CT scan (p=0.007) were independent risk factors for PM (P1 group), suggesting that patients with these characteristics should always be considered for SL.

In a previous systematic review, the use of SL provided a benefit by changing the treatment in 8.5–59.6% of cases, sparing patients from unnecessary laparotomy in 8.5–43.8% of cases^20^. These discrepancies are also partly due to the fact that the studies used different indications for performing SL. Our study revealed a considerably high percentage of therapeutic strategy change after SL (30.8%), sparing laparotomy in 24.6% of the cases and offering surgery to 6.1% of patients who were previously considered stage IVb by CT scan.

Difficulties related to the widespread adoption of SL are related to cost and its invasive risk-prone procedure^13,14,21,28^. Increasingly restricted availability of scheduling in the operating rooms for a procedure that requires general anesthesia may also play a role. Regarding the cost-effectiveness of SL, Kevin et al. reported that the expected benefit from avoiding unnecessary laparotomies may be low compared to the cost of routine use of SL. Nevertheless, it can be good if the procedure yield is high, especially in those with a high risk of occult PM suggesting a more selective practice^21^.

When comparing the survival rates of patients who underwent SL to those staged by tomography, a difference was observed in the survival curves of M0 patients by tomography vs. M0 patients by SL. Despite the absence of statistical significance, this could represent the impact of the understaging of CT scan on prognosis. A better selection of patients who are candidates for curative treatment is achieved with SL and some patients considered M0 by tomography could show occult carcinomatosis, and consequently worse prognosis and survival. A more significant number of patients should be studied to assess the statistical difference between the groups.

Despite the high accuracy of SL, of the 64 patients classified as P0 by SL, 8 (12.5%) patients presented with PM during the second surgery. Some authors reported this outcome as “false negative for SL,’, with rates ranging from 0 to 17.2%^9,12,15,23^. While this corresponds to a failure of SL, it could also represent a disease progression between SL and the second surgery. The answer, however, remains unknown.

Our study had some limitations. First, being a retrospective study, it carries an inherent selection bias in indicating patients with a greater suspicion of PM for SL. In addition, we do not perform a second SL after neoadjuvant chemotherapy. Thus, the percentage of change of conduct is higher than that found in some other studies. Also, we did not include in the study the evaluation of peritoneal lavage by cytology, since we do not have the results available for all patients. In addition, in our service, the evaluation of peritoneal lavage is performed using cytological and immunohistochemical techniques. Till present, molecular techniques such as reverse transcription-polymerase chain reaction (RT-PCR) for investigating tumor cells in the lavage are not part of our diagnostic routine. Indeed, previous studies have demonstrated improvements in the sensitivity of peritoneal washing using molecular diagnosis via RT-PCR, being also useful for predicting the peritoneal recurrence and prognosis^17^.

Notwithstanding the limitations, our study has also important strengths. It represents an 11-year period of Western tertiary single-center experience. Our findings demonstrated that SL had a significant impact on GC staging, especially in the diagnosis of PM, along with its superiority regarding conventional imaging tests. Patients with the peritoneal spread still represent a major challenge in oncology. Currently, clinical studies related to effective strategies to improve long-term survival are underway, and novel intraperitoneal chemotherapies have emerged as potential treatment options^22,29^. According to this perspective, recognizing patients with metastasis limited only to the peritoneum becomes even more important for best management^32^.

## CONCLUSIONS

Staging laparoscopy demonstrated an important role in the diagnosis of PM, contributing to the choice of the correct therapeutic strategy for the treatment of AGC. It should be considered for all advanced gastric tumors, particularly in the presence of ascites and suspected PM on CT scan.
